# Late Dr. (Mrs.) Dhanalakshmi De Sousa

**Published:** 2006

**Authors:** K. P. Dave

**Affiliations:** Retired Hon Prof and Head, Dept of Psychiatry, L.T.M.G. Medical College and Hospital, Sion, Mumbai. Also, Prof of Psychiatry, K.J. Somaiya College and Hospital, Sion

(Editor's note: Dr K.P.Dave, who worked with Late Dr De Sousa, recounts his experiences and her qualities of head and heart.)

Dr. (Mrs.) Dhanalakshmi De Sousa was my senior colleague at K.E.M. Hospital during our post-graduate studies and at L.T.M.G. Hospital during our professional career as a staff member. I had an opportunity to be with her for about 18 years.

As a Registrar at K.E.M., she appeared busy with her studies and sincere about her commitments.

At L.T.M.G. Hospital, she worked in her initial years at the Child Guidance Clinic and was later promoted to adult psychiatry.

She maintained a good relationship with patients and their relatives. She often went out of the way to seek social and economic help for the patients’ welfare.

When she became Chief after Dr. Bassa's retirement, she showed her genuine interest in the development of the Department of Psychiatry. She took great pains to get funds for equipments like EEG, ECT machines, and Behaviour Therapy instruments. She wanted to develop other diagnostic and treatment modalities which were not there during Dr. Bassa's tenure as chief. She made efforts to develop good relationship with other departments in the hospitals, and encouraged residents to do the same.

Post-graduate teaching, nurses’ education and research projects were the areas where she actively participated.

Social activities like picnics and parties, which helped to improve interpersonal relationships, were a frequent feature during her tenure as Head.

She contributed generously from her personal income to encourage under-graduates to take interest in Psychiatry. She started the ‘Dr. De Sousa Award’ for a competitive examination in Psychiatry for the under-graduates.

We were surprised to find her asking for premature retirement from L.T.MG. Hospital as a staff member at a time when she was most needed. We came to know later it was to bring up her son, Avinash, now a Psychiatrist in his own right. A courageous, and probably wise, decision to take.

We had less and less interaction with her after her retirement. But we always remembered her and quoted her during our academic post-graduate sessions in the Department.

In her sad demise, we have lost a great soul who worked for the poor and the unfortunate. And a colleague with a great affection and love for people.

May her soul rest in eternal peace.

